# Medication use by middle-aged and older participants of an exercise study: results from the *Brain in Motion* study

**DOI:** 10.1186/s12906-017-1595-5

**Published:** 2017-02-10

**Authors:** Tania Pannu, Sarah Sharkey, Grazyna Burek, Daniela Cretu, Michael D. Hill, David B. Hogan, Marc J. Poulin

**Affiliations:** 1 Department of Physiology and Pharmacology, Hotchkiss Brain Institute, Cumming School of Medicine, University of Calgary, HMRB-210, 3330 Hospital Drive NW, Calgary, AB T2N 4N1 Canada; 2 Calgary Stroke Program, Department of Clinical Neurosciences, University of Calgary, Foothills Hospital, Rm 1242A, 1403 29th Street NW, Calgary, AB T2N 2 T9 Canada; 3 Department of Medicine, Cumming School of Medicine, University of Calgary, Calgary, Canada; 4 Department of Clinical Neurosciences, Cumming School of Medicine, University of Calgary, Calgary, Canada; 5 Faculty of Kinesiology, University of Calgary, Calgary, Canada; 6 Libin Cardiovascular Institute of Alberta, University of Calgary, Calgary, Canada

**Keywords:** Natural Health Products, Prescription drugs, Geriatrics, Polypharmacy

## Abstract

**Background:**

Over the past 50 years, there has been an increase in the utilization of prescribed, over-the-counter (OTC) medications, and natural health products. Although it is known that medication use is common among older persons, accurate data on the patterns of use, including the quantity and type of medications consumed in a generally healthy older population from a Canadian perspective are lacking. In this study, we study the pattern of medication use in a sedentary but otherwise healthy older persons use and determined if there was an association between medication use and aerobic fitness level.

**Methods:**

All participants enrolled in the *Brain in Motion* study provided the name, formulation, dosage and frequency of any medications they were consuming at the time of their baseline assessment. Maximal aerobic capacity (VO_2_max) was determined on each participant.

**Results:**

Two hundred seventy one participants (mean age 65.9 ± 6.5 years; range 55–92; 54.6% females) were enrolled. Most were taking one or more (1+) prescribed medication (*n* = 204, 75.3%), 1+ natural health product (*n* = 221, 81.5%) and/or 1+ over-the-counter (OTC) drug (*n* = 174, 64.2%). The most commonly used prescribed medications were HMG-CoA reductase inhibitors (statins) (*n* = 52, 19.2%). The most common natural health product was vitamin D (*n* = 201, 74.2%). For OTC drugs, non-steroidal anti-inflammatories (*n* = 82, 30.3%) were the most common. Females were more likely than males to take 1+ OTC medications, as well as supplements. Those over 65 years of age were more likely to consume prescription drugs than their counterparts (*p* ≤ 0.05). Subjects taking more than two prescribed or OTC medications were less physically fit as determined by their VO_2_max. The average daily Vitamin D intake was 1896.3 IU per participant.

**Conclusions:**

Medication use was common in otherwise healthy older individuals. Consumption was higher among females and those older than 65 years. Vitamin D intake was over two-fold higher than the recommended 800 IU/day for older persons, but within the tolerable upper intake of 4,000 IU/day. The appropriateness of the high rate of medication use in this generally healthy population deserves further investigation.

## Background

Approximately 60% of older persons live a sedentary lifestyle and do not engage in sufficient physical activity to acquire health benefits [1]. Physical inactivity is a known modifiable risk factor for cerebrovascular disease and dementia [2, 3]. The doubling of the older population over the next 25 years will be associated with increases in the numbers of those with cognitive decline and dementia [4].

Between 1997 and 2007, Canada was second to the United States in the growth of prescription drug expenditure per capita [5]. Annual growth was 10.1%, and by the end of this period, prescription drug expenditures had become the second largest component of health care spending in Canada [5]. Heavy use of pharmaceuticals becomes particularly marked at older age ranges [6–9]. Eighty three percent of community-dwelling Canadians 65–79 years of age in 2007–2011 were taking prescription medication with 33% taking 5 or more [6]. In addition to prescribed medications, there is a widespread use of over-the-counter (OTC), dietary supplements, and alternative agents even within healthy populations [5, 8, 10, 11]. The use of complementary and alternative medicine (CAM), defined by Health Canada as being for the “diagnosis, treatment and/or prevention that complements mainstream medicine”, is very common with a large proportion of adults using natural health products. Although the rise in the use of pharmaceuticals is well known, the pattern of the combined use of the various types of medications being consumed by individuals is less well defined. Few studies have described medication use in a unique population composed of otherwise healthy middle-aged and older sedentary Canadians.

The *Brain in Motion* (BIM) study is a quasi-experimental prospective cohort study designed to determine the effect of aerobic exercise for six months on cognitive function and cerebrovascular physiology in the sedentary older adults enrolled in our study. Because of the study’s exclusionary criteria, the study population was anticipated to be healthier than the general population of Canadians of a similar age. The purpose of this study was to describe the pattern of medication use by study participants at baseline prior to the exercise intervention component of the BIM study. Furthermore, we examined whether there was an association between participants’ fitness levels and the use of medications. Given their health status, we hypothesized these participants would show lower levels of medication consumption compared with similarly aged Canadians.

## Methods

**Fig. 1:**
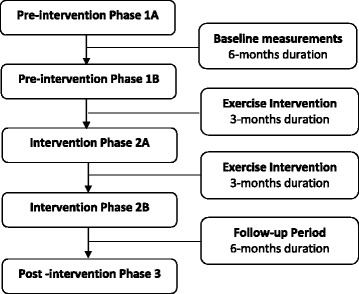
Flow chart outlining the phases of the BIM study. Medication use was obtained at the time of baseline assessment (Pre-intervention Phase 1A)

BIM is an 18-month quasi-experimental prospective cohort study examining the role of exercise on cognitive function and cerebrovascular physiology [12]. The study consisted of three six-month phases: 1) pre-intervention phase; 2) aerobic exercise intervention phase; and 3) post-intervention phase (Fig. 1) [12]. The Conjoint Health Research Ethics Board at the University of Calgary approved the study, and participants provided written informed consent prior to participating in the study. Medication use was obtained at the time of the baseline assessment (Pre-intervention Phase 1A) (Fig. 1), during which time participants were asked to list the name, formulation, dosages, and frequency of consumption of all medications and supplements that they were currently taking. These agents were then categorized as prescribed (i.e., require a prescription to be obtained from their physician) and non-prescribed, which were further sub-categorized as OTC medications (i.e., available for purchase without a prescription) as natural health products (which included vitamins and minerals, herbal remedies, homeopathic medicines, traditional medicines, or probiotics). We defined drug use intensity as the mean number of medications consumed per person. Years of education were also recorded at baseline, and defined as total years completed in a formal education program starting with primary (elementary) school.

### Recruitment and eligibility

Subjects were recruited from the City of Calgary between 2009 and 2014 through media, poster and newspaper advertisements. Potential participants provided informed written consent prior to enrollment into the study. During an initial phone interview, interested participants were assessed for their eligibility based on pre-determined inclusion and exclusion criteria, as discussed below. Potential participants who successfully completed the telephone-based evaluation, were then scheduled for a 60-min on-site assessment of study eligibility [12]. Participation in the study required approval by their attending primary care physician, and physicians affiliated with the study examined all subjects at entry to ensure their safety [12].

### Subjects

Subjects were sedentary men and women aged 55 years and older. Sedentary behaviour was defined as, engaged in less than 30 min of moderate exercise 4 days per week or 20 continuous minutes of vigorous exercise twice weekly. Inclusion criteria included: Body Mass Index of less than 35 kg/m^2^; ability to walk independently outside and up and down at least 20 stairs; non-smoker for at least 12 months; not suffering from an active cardiovascular, cerebrovascular, or pulmonary condition that would preclude their ability to safely exercise; no major surgery or trauma within the last 6 months; free from a debilitating neurological condition; and, a Montreal Cognitive Assessment score of 24 or greater [12].

### Maximal aerobic capacity

At baseline, subjects underwent assessments of fitness levels by a maximal aerobic capacity (VO_2_max) test. This was used as our measure of fitness. The VO_2_max test was conducted on a motorized treadmill and follows the Bruce protocol [13]. A plateau in oxygen uptake with increasing work rate (<2 mL/kg/min), a respiratory exchange ratio of at least 1.15, and age-predicted maximal heart rate (210-(age X 0.65)) were used as criteria for stopping the test (as recommended by the Canadian Society for Exercise Physiology [14] and American Thoracic Society [15]. Outcome measures for the VO2max test include oxygen uptake, carbon dioxide production, and ventilation (tidal volume and breathing frequency) [12].

### Data analysis

Categorical proportions were compared using Fisher’s exact of Chi2 tests. Relationships between VO_2_max and medication usage were analyzed using a generalized linear model, whereby we determined the combined effects of sex and medication usage on VO_2_max when controlling for age.

## Results

Among the 271 subjects (mean [SD] age 65.9 ± 6.5 years, 54.6% females) enrolled in the *Brain In Motion* study who completed their baseline assessment (Table 1), most subjects were taking at least one prescription drug (75.3%), natural health product (81.5%), and/or OTC drug (64.2%). The most commonly prescribed medications were cholesterol lowering agents (specifically HMG-CoA reductase inhibitors or statins) and thyroid replacement therapy (Table 2). Vitamin D was the most common natural health product consumed, while non-steroidal anti-inflammatories (NSAIDs) were the most common OTC medication reported (Table 3). The average daily vitamin D intake was 1896 IU per participant with 42% of participants taking more than 2000 IU per day and 10% above 4000 IU (Table 4).

**Table 1:** Baseline characteristics of subjects enrolled in the *Brain in Motion* study

Demographics	Number of subjects (*N* = 271)
Average age ± SD	65.9 ± 6.5
% (number) female sex	54.6% (148)
Average number of years of education ± SD	15.9 ± 2.7
Total % (number) of participants consuming 1+ prescribed medication at baseline	75.3% (204)
Total % (number) of participants consuming 1+ natural health product at baseline	81.5% (220)
Total % (number) of participants consuming 1+ over-the-counter medication at baseline	64.2% (174)

**Table 2:** Baseline prescribed medication use among participants (*N* = 271) enrolled in the *Brain in Motion* study (listed alphabetically)

Prescribed medications	% total participants (number)
Drugs used for diabetes	4.4% (12)
Anticonvulsant	3.0% (8)
Antihypertensive/ cardiovascular agents	
^b^ACE Inhibitor	8.9% (24)
Angiotensin Receptor blocker	12.5 (34)
Beta blocker	3.7% (10)
Calcium Channel blocker	10.0% (27)
Diuretic	14.0% (38)
Analgesic	
^b,a^NSAID	12.2% (33)
Opioid Analgesic	3.3% (9)
Antidepressant	14.4% (39)
Benign Prostrate Hyperplasia Agent	
Alpha blocker	2.6% (7)
Cholesterol lowering agent	
^b^HMG-CoA reductase inhibitors (Statins)	19.2% (52)
Hormonal agent	
Estrogen	9.2% (25)
Corticosteroid	7.4% (20)
Progesterone	2.6% (7)
Thyroxine	17.0% (46)
Proton Pump Inhibitor	7.4% (20)
Sleep aids	
Melatonin Receptor Agonist	3.3% (9)
Sedatives	12.2% (33)
Other	
Antispasmodic	3.3% (9)
Bisphosphonate	7.0% (19)
Prostaglandin	5.9% (16)
Other	24.7% (67)
None	24.7% (67)

^b^ *ACE* Angiotensin Converting Enzyme

*NSAID* Non-Steroidal Anti-Inflammatory Drug

*HMG-CoA* 3-Hydroxy-3-Methyl-Glutaryl-CoA

^a^These include only prescribed NSAIDs

**Table 3:** Baseline supplement, natural produce and over-the-counter medication use among participants (*N* = 271) enrolled in the brain in motion study

Natural health products	% Total participants (number)	Over-the-counter medication	% Total participants (number)
Antioxidants	4.8% (13)	Analgesic	29.9% (81)
Coenzyme Q10	7.0% (19)	Antihistamine	4.8% (13)
Digestive Enzymes	2.2% (6)	Laxative	3.3% (9)
Ginkgo	2.6% (7)	Muscle Relaxant	2.2% (6)
Ginseng	2.2% (6)	NSAID	30.3% (82)
Glucosamine	18.8% (51)	Sedative	1.8% (5)
Lutein	7.5% (20)	Other	10.0% (27)
Melatonin	1.8% (5)	None	35.8% (97)
Minerals			
Calcium	27.7% (75)		
Other (e.g. magnesium, zinc)	16.6% (45)		
Multi-vitamins	33.9% (92)		
Omega 3	37.6% (102)		
Plant Oil	4.1% (11)		
Probiotics	4.4% (12)		
Vitamins			
Vitamin D*	74.2% (201)		
Vitamin C	15.9% (43)		
Vitamin E	7.7% (21)		
B-complex	18.1% (49)		
Other (e.g. A, K)	1.8% (5)		
Other	32.5% (88)		
None	45.8% (124)		

*Includes 180 unique participants taking a vitamin D supplement and 21 taking multivitamins not on a separate vitamin D supplement

**Table 4:** Vitamin D analysis in 201 participants on Vitamin D supplements. This analysis does not include dietary intake

Vitamin D analysis, *N* = 201 (per day)	
Recommended intake (IU)*	800
Average intake (IU)	1896.3
Median intake (IU)	1400
Minimum dosage (IU)	200
Maximum dosage (IU)	12000
% of subjects taking > 800 IU (n)	79.1 (159)
% of subjects taking ≥ 2000 IU (n)	41.8 (84)
% of subjects taking ≥ 4000 IU (n)	9.5 (19)

* *IU* International Units

More females than males were taking one or more OTC medications (70% women v 57% men). Natural health products (58% women v 42% men) and prescribed medications (78% women v 72% men) use was similar between the sexes. Older subjects (age > 65 years) were more likely to be taking at least one medication in all three categories and had a higher prescribed drug use intensity (mean number of medications consumed per person) (Table 5). When controlling for age, men and women taking more than two prescribed or OTC medications were less physically fit, as determined by their VO_2_max measurements (Table 6).

**Table 5:** Number (%) of people taking prescribed medications according to the age group and number of medications per person (*N* = 271)

Number of prescribed medications	Age group		
	≤65 (*N* = 127) % (number)	>65(*N* = 144) % (number)	*P*-value*
0	30.7 (39)	19.4 (28)	0.019
1	22.8 (29)	22.2 (32)	
2	20.5 (26)	20.8 (30)	
3	14.2 (18)	13.2 (19)	
4	7.9 (10)	8.3 (12)	
5 or more	3.9 (5)	16.0 (23)	

*Fisher’s exact test

**Table 6:** Physical fitness analysis using VO2_max_ levels in 261 participants consuming OTC and prescription medication

Medication Count	All Participants			Female			Male		
	*N*	*VO2*_*max*_ *Mean(SD)*	*P-value*	*N*	*VO2*_*max*_ *Mean(SD)*	*P-value*	*N*	*VO2*_*max*_ *Mean(SD)*	*P-value*
0	57	28.36(6.02)*	-	24	24.59(5.17)	-	33	31.10(5.08)	-
1	65	27.23(5.63)*	>0.05^a^	35	24.6(4.94)	>0.05^a^	30	30.30(4.82)	>0.05^a^
2	53	25.28(4.69)	0.03^b^	29	23.55(4.04)	0.04^b^	24	27.36(4.63)	0.04^b^
>3	86	23.72(4.36)*	<0.001^c^	53	22.26(3.67)	0.005^c^	33	26.06(4.40)	<0.001^c^

Sex difference: *P < 0.05

*N* Number of participants in each category

*P*-values: ^a^difference between 0 medication and >3 medications; ^b^difference between 1 medication and >3medications; ^c^difference between 2 medications and >3 medications

## Discussion

This study presents data on the pattern of medication use in sedentary but otherwise generally healthy middle-aged and older adults. According to the Chief Public Health Officer’s Report on the State of Public Health in Canada 2010, approximately one-quarter (23%) of Canadian older persons (i.e., 65 or more years of age) suffered from cardiovascular disease and over 4% from stroke. Those with active cardiovascular or debilitating neurological conditions were excluded from entry into our study, which also recruited a number of individuals less than 65 years of age [16]. While 62% of Canadian seniors reported being in very good functional health, nearly one-quarter of them had difficulties with activities such as walking, communicating, seeing, and climbing stairs [16]. BIM participants had to be able to function independently without difficulties. They were able to perform moderate physical activity three times a week, for the 6-month intervention period, and also underwent demanding physiological testing (such as VO_2_max tests) a number of times during the 18-month study.

Most subjects (75.3%) were taking at least one prescription drug at baseline (most commonly used were cholesterol lowering agents and L-thyroxine) (Table 2). Canadians in general are heavy users of prescribed medications. A total of $27,734 million (or $795.32 per capita) was spent on prescription drugs nationally in 2012 [6, 17]. In 2012 the most common prescription medication used by older persons in Canada were statins (46.6%), followed by angiotensin-converting-enzyme (ACE) inhibitors (28.4%) and proton pump inhibitors (27%) [18]. While BIM participants commonly were taking statins, their rate of use (19.2%) was less than half of that reported for the general older population (Table 2). Use of ACE inhibitors (8.9%) and proton pump inhibitors (7.4%) was also substantially lower than that reported for the general older Canadian population [9]. This is consistent with our impression that study participants were generally healthier than the older Canadian population. Similar to other studies female sex and older age were associated with a greater intensity of prescribed medication use [6–9].

A majority of subjects were taking at least one natural health product (81.5%) or OTC medication (64.2%) (Table 1). NSAIDs were the most commonly used OTC medication (30.3%) in our study. One of the commonest reason for supplement use is a desire to improve or maintain overall health [19]. There are limited data to support the health benefits of supplements and natural products in generally healthy individuals, and there is a potential, albeit small, of risk of harm with their use. Adverse drug effects and drug-drug interactions can occur [20–33]. Supplements can reduce the efficacy of specific medications [10, 22–25, 28–37] while the consumption of multiple agents concurrently is associated with a higher risk of non-adherence [35]. Although prescription medications have a higher likelihood of side effects, there is greater regulatory oversight. Since these medications require a prescription, physicians and pharmacists should be informing patients of potential side effects and monitoring for them. With OTC medications the attending physician may be unaware of their use (which is commonly the case) [29] with no monitoring for side effects and assessment for potential interactions. Consumption of these agents tends to increase with age [36]. The use of medications (prescription and OTC) is associated with a decrease in the fitness levels of participants (Table 6). Because of the observational nature of this study no conclusions about a causal relationship can be made. Possibly the presence of various clinical conditions alone or in combination with other ones are associated with both medication use and worse fitness levels.

Nearly three-quarters of our study population were taking vitamin D. The average daily intake was 1896 IU per participant with nearly half consuming more than 2000 IU per day and 10% above 4000 IU (Table 4). Nearly half (47.5%) of the Canadian population is reportedly taking supplemental vitamin D [38] at a median dose of 400 IU/day [39]. Factors reported to be associated with a greater likelihood of use included increasing age, female sex, English language, diagnosis of osteoporosis, perceived lactose intolerance, and university education [38]. In postmenopausal women and men above 50, vitamin D and calcium supplementation might lead to increased bone density and reduce the risk of falls and hip and non-vertebral fractures [40] though these claims are controversial [41, 42]. Adults over 50 years of age are at a moderate risk of vitamin D deficiency. Vitamin dosages as high as 2000 IU/day can safely be given without the need for any monitoring [39, 43]. While some feel doses up to 4000 IU/day are safe [44], others believe that doses above 2000 IU/day should be supervised because of potential risks of hypercalcemia and renal dysfunction [40, 43].

Our study had a number of limitations. We did not know the indications for the medications taken, or whether they were effective for the indication of their use. We were unable to determine whether OTC medications were recommended by a physician or taken without their advice or knowledge. We did not assess adherence. Together, this means that we cannot comment on the appropriateness of the medications being taken. Our emphasis was on describing overall use rather than understanding their indications. Finally, given the strict inclusion criteria for the Brain in Motion study, our findings relating to the medication use in this group, may not be representative of the general population.

## Conclusion

This study showed high medication use (prescribed, OTC, and natural health products) in a generally healthy group of middle-aged and older Canadians. Consumption was higher in females, and amongst those older than 65 years. Higher medication usage was associated with a lower fitness level. Rate of medication use was lower than that of the general older Canadian population. The appropriateness of this the high rate of medication use in this generally healthy population deserves further investigation.

## Abbreviations

BIM: Brain in motion
CAM: Complementary and alternative medicine
NSAID: Non-steroidal anti inflammatory
OTC: Over the counter
